# Synthetic colloid resuscitation in severely injured patients: analysis of a nationwide trauma registry (TraumaRegister DGU)

**DOI:** 10.1038/s41598-018-30053-0

**Published:** 2018-08-01

**Authors:** Peter Hilbert-Carius, Daniel Schwarzkopf, Konrad Reinhart, Christiane S. Hartog, Rolf Lefering, Michael Bernhard, Manuel F. Struck

**Affiliations:** 1 Department of Anesthesiology, Intensive Care and Emergency Medicine, Pain Therapy; Bergmannstrost Hospital Halle, Halle, Germany; 2 Center for Sepsis Control and Care, Jena University Hospital, Jena, Germany; 3 Department of Anesthesiology and Intensive Care Medicine, Jena University Hospital, Jena, Germany; 4 Klinik Bavaria Kreischa, Kreischa, Germany; 5 Institute of Research in Operative Medicine (IFOM), University Witten-Herdecke, Campus Cologne Merheim, Cologne, Germany; 6 Emergency Department, University Hospital Duesseldorf, Duesseldorf, Germany; 7 Department of Anesthesiology and Intensive Care Medicine; University Hospital Leipzig, Leipzig, Germany

## Abstract

The purpose of this study was to investigate the efficacy and safety of synthetic colloid resuscitation among severely injured patients. Fluid resuscitation of trauma patients of a nationwide trauma registry was analysed between 2002 and 2015. Effects of synthetic colloid resuscitation in the pre-hospital setting and emergency department on renal failure, renal replacement therapy and multiple organ failure were analysed among patients with ≥2 days intensive care unit stay, and in-hospital mortality was analysed among all patients. 48,484 patients with mean age of 49 years and mean injury severity score of 23 points were included; 72.3% were male and 95.5% had blunt trauma. Risk-adjusted analyses revealed that patients receiving >1,000 ml synthetic colloids experienced an increase of renal failure and renal replacement therapy rates (OR 1.42 and 1.32, respectively, both *p* ≤ 0.006). Any synthetic colloid use was associated with an increased risk of multiple organ failure (*p* < 0.001), but there was no effect on hospital mortality (*p* = 0.594). Between 2002 and 2015 usage of synthetic colloids dropped, likewise did total fluid intake and usage of blood products. The data from this analysis suggests that synthetic colloid resuscitation provides no beneficial effects and might be harmful in patients with severe trauma.

## Introduction

### Purpose

Synthetic colloid infusion solutions have been developed to increase haemodynamic stabilization effectively and economically. One of the most frequently used synthetic colloid worldwide is hydroxyethyl starch (HES)^1^. Concerns about the use of starches grew after three multicentre randomised controlled trials (RCTs) found that HES was associated with a higher risk of acute kidney injury and bleeding in patients with sepsis or critical illness^2^ and more deaths in patients with sepsis^3,4^ when compared with crystalloids. The trials prompted the German Federal Institute for Drugs and Medical Devices to ask the European Medicines Agency (EMA) to review the use of HES in 2012. The EMA committee recommended suspending all use of HES. However, after the United Kingdom unilaterally removed HES from the shelves, a second EMA review was triggered. A second assessment committee confirmed that HES solutions must not be used in critically ill patients or those with sepsis and burn injuries but allowed their continued use in patients with hypovolaemia due to acute blood loss within the first 24 hours after elective surgery or trauma^5^. This decision was based on “some reassurance that the risks of mortality and renal injury in surgical and trauma patients may be lower than those in critically ill and [septic] patients”^6^. However, there is only one small prospective RCT in trauma patients^7,8^. In general, published studies on synthetic colloid resuscitation in the trauma population have methodological weaknesses due to low sample sizes, limited observation periods, incomparable study groups, missing control groups, inconsistent definitions and study endpoints with low clinical relevance^5,7,9–14^. Moreover, in early 2017 the US based patient advocacy group Public Citizen filed a petition with the U.S. Food and Drug Administration (FDA) requesting that the FDA ban HES solutions in the U.S, because of the “unique risks of HES solutions” and the fact that they offer “no benefit over the other types of intravenous solutions on the market”^15^. The EMA committee requested large randomised post-marketing clinical trials from HES manufacturers to establish the efficacy and safety of HES in perioperative and trauma populations^16,17^. Currently, such data is not yet available. Although the final decision by the European Commission is still pending, the medicines regulatory body of the European Union member states (CMDh) agreed in early 2018 with the EMA’s Pharmacovigilance Risc Assessment Committee recommendation that HES solutions should be suspended^18^. Thus, the aim of this retrospective registry study was to further elucidate the risk benefit ratio of synthetic colloids in a large registry that comprises severely injured patients.

## Results

During the study period, 199,977 patients were recorded in the TraumaRegister DGU (TR-DGU) database. Of 119,530 patients of age >16 years, injury severity score (ISS) ≥9, direct admission from the scene of accident, and without early referral to another hospital, 48,484 (41%) had standard data documentation and were included for the analyses. The mean age was 48.5 ± 20.5 years (mean ± standard deviation), mean ISS was 22.7 ± 12.3 points. The majority of patients were male (72.3%) and had sustained blunt trauma (95.5%). The trauma mechanisms included motor vehicle accidents (27.3%), falls from >3 m height (18.1%), falls from <3 m height (15.6%), motorcycle accidents (15.2%), bicycle accidents (8.3%), pedestrian accidents (7.4%) and patients who could not be classified into one of these groups (8.0%). Intensive care unit (ICU) length of stay was in median [1^st^ quartile, 3^rd^ quartile] 4 [2, 13] days, hospital length of stay was 16 [9, 28] days.

**Figure 1:**
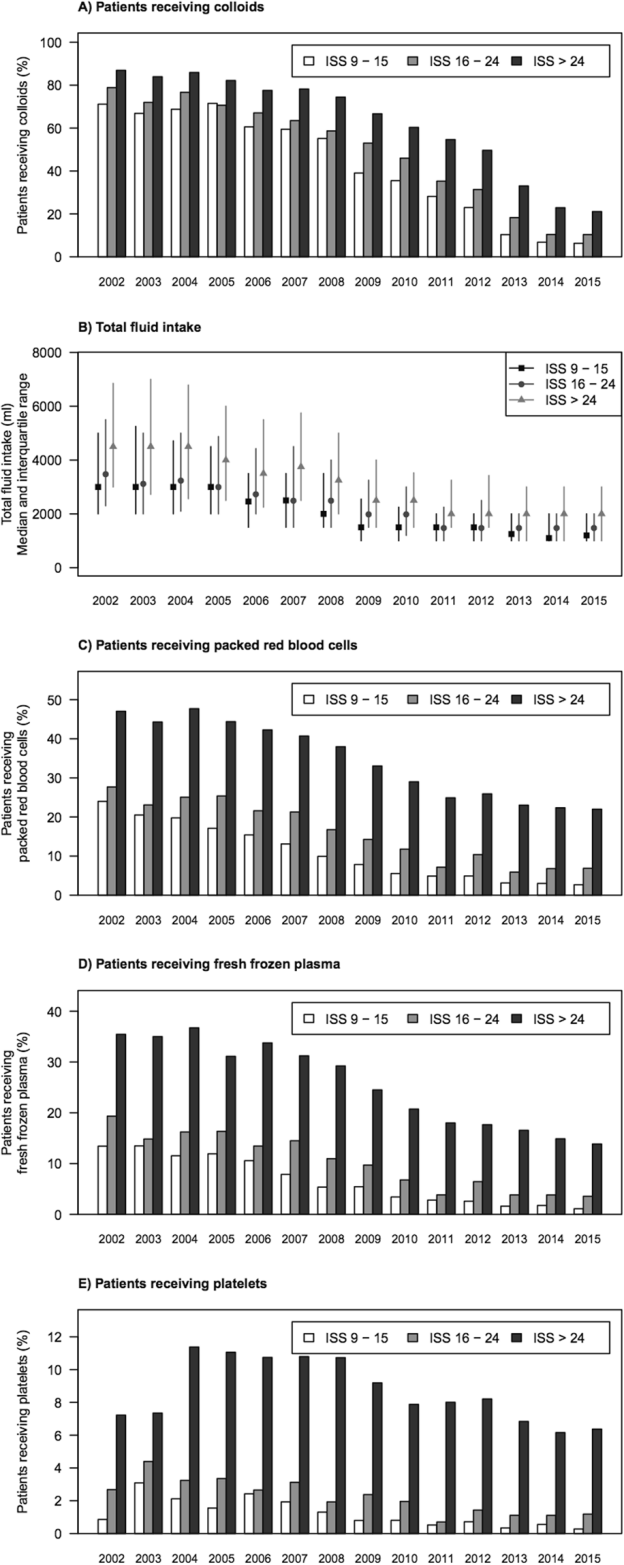
Fluid resuscitation and transfusion of blood products over time, stratified by injury severity score (ISS).

The demographic data of the study cohort and laboratory results at emergency department admission are shown in Table 1. Table 2 provides fluid resuscitation volumes and transfusion of blood products. Patients receiving higher synthetic colloid volumes received more blood products. Synthetic colloid resuscitation decreased considerably over the course of the study period (Fig. 1A). Likewise, total fluid intake, as well as use of red blood cells, fresh frozen plasma and platelet concentrates decreased (Fig. 1B–E).

**Table 1:** Baseline characteristics, laboratory results, and length of stay.

Parameter	All patients n = 48,484	Crystalloids only, n = 28,942	Synthetic colloids ≤500 ml, n = 7,416	Synthetic colloids 500–1,000 ml, n = 5,686	Synthetic colloids >1,000 ml, n = 6,440
Age, years	48.5 ± 20.6	51.4 ± 20.7	46.4 ± 20.3	44.5 ± 19.6	41.3 ± 18.1
Male gender	34,943 (72.3%)	20,436 (70.8%)	5,458 (74%)	4,153 (73,4%)	4,896 (76.3%)
Blunt trauma	45,048 (95.5%)	27,060 (96.7%)	6,848 (94.7%)	5,270 (94.5%)	5,870 (92.3%)
Injury Severity Score, points	22.7 ± 12.3	20.2 ± 10.5	24.5 ± 12.9	26.4 ± 13.7	29 ± 14.2
Glasgow coma scale (pre-hospital)	14 [9, 15]	14 [11, 15]	14 [7, 15]	13 [7, 15]	13 [6, 15]
SBP at ED ad- mission, mmHg	130 [110, 147]	132 [120, 150]	120 [108, 140]	120 [100, 140]	115 [99, 130]
Heart rate at ED ad- Mission, 1/min	88 [76, 100]	85 [75, 98]	89 [77, 100]	90 [80, 102]	92 [80, 110]
Shock at ED admission (SBP ≤90 mmHg)	4,885 (10.5%)	1,637 (5.9%)	911 (12.7%)	916 (16.6%)	1,421 (22.8%)
Expected mortality (%) based on RISC II score	12.8 ± 23.6	10.4 ± 21.3	14.2 ± 25	16.2 ± 26.4	19 ± 27.5
**ED admission laboratory results**					
Haemoglobin, g/dl	12.9 [11.1, 14.3]	13.5 [12.1, 14.7]	12.5 [10.8, 13.9]	11.9 [10, 13.5]	10.7 [8.7, 12.6]
Platelet count, Gpt/L	211 [168, 257]	218 [177, 263]	208 [165, 255]	198 [155, 249]	186 [142, 235]
Activated partial thromboplastin time (aPTT), sec	28 [25, 32]	27 [24.3, 31]	28.5 [25, 33]	29.4 [26, 35]	32 [27, 41]
Prothrombin time (Quick’s value), %	87 [72, 99]	91 [79, 100]	84 [70, 96]	80 [64, 93]	71 [54, 87]
International normalized ratio (INR)	1.09 [1, 1.2]	1.05 [1, 1.14]	1.1 [1.01, 1.23]	1.14 [1.04, 1.30]	1.23 [1.09, 1.48]
Lactate, mmol/l	2 [1.3, 3.2]	2 [1.3, 3]	2.01 [1.3, 3.28]	2.2 [1.4, 3.7]	2.4 [1.5, 4.2]
Base excess, mmol/l	−1.7 [−4.2, 0.5]	−1 [−3.1, 1]	−2.3 [−4.8, −0.1]	−2.9 [−5.6, −0.6]	−4 [−6.9, −1.5]
**Length of stay**					
ICU length of stay, days	4 [2, 13]	3 [1, 9]	6 [2, 15]	7 [3, 17]	10 [4, 21]
Hospital length of stay, days	16 [9, 28]	14 [8, 23]	19 [10, 30]	22 [12, 35]	26 [13, 42]

Descriptive statistics given as mean ± standard deviation, median [1^st^ quartile, 3^rd^ quartile], or n (%). ED; emergency department, SBP; systolic blood pressure, RISC II score; Revised Injury Severity Classification score revision 2, ICU; intensive care unit.

**Table 2:** Fluid resuscitation and transfusion of blood products until intensive care unit admission.

Fluid volumes	All patients n = 48,484	Crystalloids only, n = 28,942	Synthetic colloids ≤500 ml, n = 7,416	Synthetic colloids 500–1,000 ml, n = 5,686	Synthetic colloids >1,000 ml, n = 6,440
Total fluids pre-hospital, ml	1,044 ± 780	748 ± 513	1,143 ± 662	1,428 ± 763	1,923 ± 1,034
Crystalloids until ICU, ml	2,074 ± 1,650	1,619 ± 1,215	2,183 ± 1,615	2,526 ± 1,742	3,600 ± 2,173
Colloids until ICU, ml	472 ± 805	0 ± 0	482 ± 70	979 ± 77	2,132 ± 940
pRBC given (%)	17.4%	5.2%	18.5%	30.5%	59.1%
No. of units, if given*	6.7 ± 7.8	5.1 ± 5.9	5.3 ± 5.8	5.9 ± 5.9	8.8 ± 9.2
FFP given (%)	11.9%	3.0%	11.6%	20.2%	45.3%
No. of units, if given*	8.0 ± 8.0	6.4 ± 6.3	6.5 ± 6.0	6.8 ± 6.0	9.4 ± 6.3
Platelets given (%)	3.9%	1.1%	3.2%	5.9%	16.0%
No. of units, if given*	2.6 ± 2.7	2.4 ± 2.3	2.4 ± 2.2	2.3 ± 1.9	2.8 ± 3.2

Descriptive statistics given as mean ± standard deviation. ICU; intensive care unit, pRBC; packed red blood cells, FFP; fresh frozen plasma.

### Effects of synthetic colloid dosage on outcomes

Table 3 presents the effects of synthetic colloid dosage on the study outcomes given as descriptive statistics, univariate odds ratios and adjusted odds-ratios of logistic regression analyses controlling for the effects of risk factors and clustering. Detailed results of the multivariate analyses are given in Tables S1–S4. Among the study cohort, 36,330 patients with an ICU length of stay (LOS) >2 days were the basis for the analysis of renal replacement therapy, renal failure, and multiple organ failure. From these, a total of 1,256 patients (3.5%) underwent renal replacement therapy and 1,785 (4.9%) developed renal failure. Patients in all three synthetic colloid groups had higher rates of renal replacement therapy and renal failure than patients in the crystalloid group (all *p* ≤ 0.022 in univariate analyses). After adjustment for risk factors, only patients who received >1,000 ml synthetic colloids had significantly higher rates of renal replacement therapy (odds ratio 1.42 [95% confidence interval 1.11–1.82]; *p* = 0.006) and higher rates of renal failure (odds ratio 1.32 [1.12–1.57]; *p* = 0.001) than patients who received only crystalloids. The effects of synthetic colloid resuscitation on renal replacement therapy were not different in patient subgroups stratified by age (Fig. S1). A total of 10,026 patients developed multiple organ failure (27.6%). After adjustment for risk factors and clustering, patients in all synthetic colloid groups presented significantly higher rates of multiple organ failure than those receiving only crystalloids; odds ratios were 1.36 [1.24–1.5] in the <501 ml colloid group; 1.39 [1.27–1.53] in the 501–1,000 ml colloid group and 1.40 [1.23–1.6] in the >1,000 ml colloid group, all *p* < 0.001. Table S5 shows the rates and adusted odds ratios for different types of organ failures according to fluid therapy subgroups.

**Table 3:** Effect of synthetic colloid dosage on outcomes.

Outcome	Rates: n (%)	Univariate OR (95% CI)	p-value	Adjusted OR (95% CI)^a^	p-value
Renal replacement therapy^b^			<0.001		0.033
Crystalloids only (reference)	512 (2.5%)	1	—	1	—
Synthetic colloids ≤500 ml	216 (3.6%)	1.43 (1.22, 1.68)	<0.001	1.17 (0.97, 1.42)	0.105
500–1,000 ml	186 (3.9%)	1.54 (1.3, 1.83)	<0.001	1.10 (0.90, 1.34)	0.366
>1,000 ml	342 (6.3%)	2.56 (2.22, 2.94)	<0.001	1.42 (1.11, 1.82)	0.006
Renal failure^b^			<0.001		0.002
Crystalloids only (reference)	803 (4.0%)	1		1	
Synthetic colloids ≤500 ml	280 (4,7%)	1.18 (1.02, 1.35)	0.022	1.02 (0.9, 1.17)	0.746
500–1,000 ml	256 (5.3%)	1.35 (1.17, 1.56)	<0.001	1.05 (0.90, 1.17)	0.549
>1,000 ml	446 (8.2%)	2.14 (1.9, 2.41)	<0.001	1.32 (1.12, 1.57)	0.001
Multiple-organ-failure^b^			<0.001		<0.001
Crystalloids only (reference)	4,388 (21.8%)	1	—	1	—
Synthetic colloids ≤500 ml	1,844 (30.8%)	1.59 (1.49, 1.7)	<0.001	1.36 (1.24, 1.5)	<0.001
500–1,000 ml	1,599 (33.3%)	1.79 (1.67, 1.91)	<0.001	1.39 (1.27, 1.53)	<0.001
>1,000 ml	2,195 (40.3%)	2.41 (2.26, 2.57)	<0.001	1.40 (1.23, 1.6)	<0.001
Hospital mortality^c^			<0.001		0.594
Crystalloids only (reference)	3,021 (10.4%)	1	—	1	—
Synthetic colloids ≤500 ml	956 (12.9%)	1.27 (1.18, 1.37)	<0.001	0.96 (0.86, 1.07)	0.473
500–1,000 ml	823 (14.5%)	1.45 (1.34, 1.58)	<0.001	0.99 (0.87, 1.12)	0.829
>1,000 ml	1,112 (17.3%)	1.79 (1.66, 1.93)	<0.001	1.06 (0.91, 1.24)	0.485

OR; odds ratio, CI; confidence interval.

^a^Adjusted odds ratios result from multiple logistic regression analyses using generalized estimating equations with an exchangeable covariance matrix controlling for the following covariates: Revised Injury Severity Classification score, version 2, variables (Abbreviated Injury Scale worst injury, second-worst injury, head injury, age, gender, mechanism (penetrating vs. blunt), motor function, pupil reactivity, blood pressure, cardiopulmonary resuscitation, International Normalized Ratio, haemoglobin, base deficit; all variables categorized), red blood cell transfusion, transfusion of more than 10 packs of red blood cells, infusion of more than 4,000 ml fluids, treatment until/after 2011. Detailed results given in Suppl. Tables 1–4.

^b^Analyses conducted among 36,330 patients with an ICU stay ≥2 days.

^c^Analyses conducted among all 48,484 included patients.

Effects of synthetic colloid resuscitation on hospital mortality were investigated among all included 48,484 patients. After adjustment for risk factors, mortality rates showed no significant differences between crystalloid only and synthetic colloid groups (*p* = 0.594) (Table 3).

## Discussion

The main findings of this analysis of early fluid resuscitation in severely injured patients from a nationwide trauma registry show that synthetic colloid resuscitation of more than 1,000 ml was associated with an increased need for renal replacement therapy and synthetic colloid use at any dose was associated with an increased incidence of multiple organ dysfunction. However, risk adjusted hospital mortality rates did not differ between patients receiving additional synthetic colloid resuscitation or only crystalloids.

Our data also shows that that the adverse effects of synthetic colloids on renal function and coagulation did not differ between age groups, in contrast to findings of previous smaller studies^7,10–13^. Furthermore, synthetic colloid use decreased continuously since 2008, and this decrease was associated with a parallel decrease in overall fluid intake.

Increased incidence of kidney injury was also observed in other smaller trauma cohort studies^12,13^ and in a large observational study of patients receiving non-cardiac surgery^19^. A meta-analysis on the impact of HES 130/04 on the need for renal replacement therapy identified an increased risk in surgical patients^20^. These were consistent with findings from RCTs in sepsis^3,4^ and critically ill patients^2^ and from a prospective cohort study on synthetic colloids in cardiac surgery patients^21^. Studies with longer follow up periods might be necessary to show mortality effects of synthetic colloids.

The suggested fluid-sparing effect of colloid resuscitation in the range of a 3 to 4-folds lower fluid requirement has been refuted by large RCTs^2–4,22^. The fluid ratios in these trials ranged between 1:1 and 1:14^2–4,22^. Our data showed that the decrease in use of synthetic colloids over time was not accompanied by an increase in overall fluid intake. Moreover, transfusion of red blood cells, platelets and fresh frozen plasma consistently decreased in all severity of injury groups along with the decrease in use of synthetic colloids. Patients receiving higher volumes of synthetic colloids also received more blood products. We cannot infer if there is a causal link between synthetic colloids dosage and blood products since both are influenced by the unmeasurable factor blood loss. However, synthetic colloid administration is associated with coagulopathy and increases the need for red blood cells transfusion, as was consistently shown in large RCTs that compared crystalloids to HES in sepsis and critically ill patients^2–4^. Increased risk of bleeding after HES administration was also found in meta-analyses of RCTs in cardiac surgery patients and in the perioperative period^23,24^. Furthermore, there is strong evidence for prolonged bleeding times and decrease in clot firmness from *in-vitro* studies^25^ and evidence of increased bleeding and mortality in animal studies^26–28^.

The mechanisms of these adverse effects have been attributed to the interference of synthetic colloids with many components of the coagulation system, e.g., inducing acquired von Willebrand disease, activating platelets^29–31^, prolonging tissue storage and directing cell toxicity^32,33^. Tissue uptake may also account for the overall higher incidence of multiple organ dysfunction that we observed in the synthetic colloid group of this cohort of trauma patients. Furthermore, numerous studies have demonstrated that the adverse effects of starches are independent of molecular size, degree of substitution and starch source, e.g., potatoes or maize^2–4^. It is well established that the adverse effects of HES are dose-related and depend on the median cumulative dose^4,19,21^. Thus, the EMA considers the adverse effects of HES administration as a class effect^34^. In their recent decision to limit the use of HES, the EMA and the FDA reduced the recommended HES dose from 50 ml/kg daily to 30 ml/kg once in 24 hrs. In our study, the median cumulative dose of synthetic colloids was approximately 26 ml/kg. At this dosage, there was no effect on hospital mortality rates but patients more frequently needed renal replacement therapy and transfusion of red blood cells. These findings are similar to the findings in the CHEST trial which investigated starch use in intensive care patients. Of note, in this trial the median cumulative dose was only 18 ml/kg^2^. This suggests that the currently recommended maximal HES dose might still be harmful for patients. Presently there are no data from high-quality trials showing that 6% HES130 improved any patient-important outcomes, while there are consistent signals for harm^35^. Our findings are in line with the findings of adverse effects of synthetic colloids that result from large RCTs, systematic reviews, *in vitro* studies and animal studies. There is no evidence why the adverse effects that have been observed in sepsis and critically ill patients should not similarly apply to surgical and trauma patients. Likewise, there is no evidence of benefits of synthetic colloid use in any indication compared with cheaper and safer alternatives. Moreover, human albumin as natural colloid is also not an appropriate alternative for trauma resuscitation due to lack of beneficial effects, limited availability and higher costs^36–40^.

Registry studies have known limitations, and all findings should be interpreted with caution. Data quality and completeness of the documented parameters are usually lower in registries than in prospective clinical studies. For example, the TR-DGU did not include information regarding fluid use after ICU admission. Thus, it may be possible that patients in all groups received synthetic colloids during the course of their ICU stay. Furthermore, the parameters of this database do not allow the calculation of more sophisticated measures of acute kidney injury such as the Risk, Injury, Failure, Loss, End stage (RIFLE) score and the Acute Kidney Injury Network (AKIN) score. We cannot rule out that other covariates than those we used controlling for imbalances in the multiple logistic regression analyses may have also contributed to the unfavourable patient-relevant outcomes that we have observed in the synthetic colloid groups of this analysis. It might be possible that the administration of a synthetic colloid is a surrogate marker of sicker patients (e.g., higher rates of shock at emergency department admission of patients who received < 501 ml HES compared to patients who received crystalloids only). These difference seem unlikely to be due to small amounts of HES. Another weakness of our study was that the TR-DGU discriminated only among crystalloid, synthetic colloid and hypertonic/hyperoncotic solutions and not the type of crystalloid and synthetic colloid. However, in the German emergency medical service, the most common synthetic colloid solution used is HES^40^. Human albumin is not used for trauma resuscitation, and most crystalloids used are balanced solutions^36,39^.

We do not know to which degree the observed overall decrease in fluid intake over time, and the almost parallel reduction in transfusion of red blood cells, fresh frozen plasma and platelet concentrates was caused by the decrease in the use of synthetic colloids over time^36,37^. Other factors such as a shortening of the pre-hospital time intervals, more restrictive fluid administration and an improved practice of permissive hypotension^38,39^ may have contributed to the decrease in fluid intake and transfusion requirements. In addition, a more restrictive indication for red blood cells transfusion by a lower haemoglobin threshold as trigger for blood transfusion may have also played a role in this development.

## Conclusions

The findings of this study underline the concerns that the adverse effects of synthetic colloids found in other patient populations might also apply to trauma patients. These adverse effects on renal function were observed at cumulative doses below the currently recommended dose limit of 30 ml/kg. The results of this study also support national and international guideline recommendations, which recommend the use of crystalloids in trauma patients.

## Methods

### Data source

The TraumaRegister DGU (TR-DGU) of the German Trauma Society (Deutsche Gesellschaft für Unfallchirurgie, DGU) was founded in 1993. The aim of this multicentre database is to create a pseudonymised and standardised documentation of patients suffering from major trauma. Data are collected prospectively at four consecutive time points from the site of the accident until hospital discharge: (A) pre-hospital emergency medical service, (B) emergency department and the initial surgery, (C) ICU, and (D) hospital discharge. The documentation includes detailed information on demographics, patterns of injury, comorbidities, pre-hospital and in-hospital management, ICU course, relevant laboratory findings (including data on transfusions), and the outcomes of each individual. The inclusion criterion is hospital admission through the emergency department, with subsequent ICU treatment or hospital admission with vital signs and death before ICU admission. Beside German hospitals also hospitals from 7 other European countries participate in the TR-DGU, but include only about 11% of the total patients. Further information regarding the TR-DGU is available at www.traumaregister-dgu.de.

### Sample

Only German trauma centers were considered for this study. The criteria for patient inclusion were ISS ≥9 points, age ≥16 years, direct admission from the scene of the accident to a trauma centre, no early referral to another hospital, standard documentation (availability of information regarding acute kidney injury, renal replacement therapy, multiple organ failure, red blood cell transfusion, and mortality), from 2002–2015, and fluid resuscitation in the pre-hospital emergency medical service and/or emergency department phase.

### Measurements

To investigate dose-related effects of synthetic colloid infusions, the study cohort was divided in four groups: (1) patients receiving only crystalloids, (2) patients receiving additional synthetic colloid volumes ≤500 ml, (3) patients receiving additional synthetic colloid volumes 501–1,000 ml, and (4) patients receiving additional synthetic colloid volumes >1,000 ml. The type of synthetic colloid was not specified. However, in Germany, colloid fluid resuscitation in the pre-hospital emergency medical service and emergency department phase is performed only with synthetic colloids, which are primarily HES solutions^40^. Furthermore, the pre-hospital transfusion of blood products is not performed in Germany.

The primary study end points were renal replacement therapy and renal failure during hospital stay. The secondary end points were multi-organ-failure, and hospital mortality. In the TR-DGU, organ failure was documented according to the following criteria^41^: sequential organ failure assessment (SOFA) scores ≥3 points of each organ system or SOFA central nervous system: Glasgow coma scale (GCS) <9; SOFA cardiovascular: requirement of adrenaline, noradrenaline or dobutamine; SOFA liver: bilirubin >6 mg/dL; SOFA kidney: creatinine >3 mg/dL or <500 ml urine output/24 h; and SOFA pulmonary: PaO_2_/FiO_2_-ratio <200. Multiple organ failure was defined as failure of two or more organ systems for at least two days according to the TR-DGU criteria outlined above.

### Statistical analyses

Fluid therapy groups were compared regarding patient and treatment characteristics using standard descriptive statistics. To investigate secular changes in fluid resuscitation therapy during the study period, annual descriptive data were plotted for total fluid volume, use of synthetic colloids, use of red blood cells, fresh frozen plasma, and platelet concentrates. To control for possible bias these plots were stratified by ISS severity: moderate (ISS 9–15), severe (ISS 16–24) and critical (ISS ≥ 25).

To test the effects of synthetic colloid dose on renal failure, renal replacement therapy, multiple organ failure, and hospital mortality, logistic regression models were calculated. Analyses regarding hospital mortality were performed based on all included patients. Analyses regarding renal failure, renal replacement therapy and multiple organ failure were performed among patients with at least two days of ICU stay to allow for appropriate calculation according to the definition of TR-DGU based on the respective categories of the SOFA score. Variables of the Revised Injury Severity Classification score, version II (RISC II), were included in the models to control for typical risk-factors for patients with injuries^41^. RISC II variables control for missing values by including them as subcategories. Additionally, transfusion of red blood cells and infusion of more than 4,000 ml of fluids were included to control for possible iatrogenic coagulation impairment and bleeding complications. Year of treatment ≥2012 was included to control for secular changes associated with the introduction of German S3 guidelines on the management of severely injured patients^39^. To control for effects of clustering of patients in hospitals, logistic regressions were calculated using generalized estimating equations with an exchangeable covariance matrix^42^. Statistical tests were performed at two tailed significance levels of *p* ≤ 0.05. All computations were performed using SPSS 18 (IBM, Armonk, NY, USA), figures were created using R (R Development Core Team, Vienna, Austria).

#### Ethics approval and consent to participate

The need of ethical approval was waived by the Ethical Review Board of the Medical Association of Saxony-Anhalt, Halle, Germany (project ID 16/17–2017). The present study was in line with the publication guidelines of the TraumaRegister DGU and was registered as TR-DGU project ID 2013–068. Consent to participate was not applicable owing to the retrospective nature of the study.

#### Availability of data and material

The datasets generated and/or analysed during the current study are not publicly available due to copyright regulations of the TraumaRegister DGU of the German Trauma Society but are available from the corresponding author on reasonable request.

## Electronic supplementary material

Tables S1–5 and Figure S1
