# Supplementary material for: Synthetic colloid resuscitation in severely injured patients: analysis of a nationwide trauma registry (TraumaRegister DGU)
Source: Sci Rep. 2018 Aug 1;8:11567. doi: 10.1038/s41598-018-30053-0 (PMC6070577; doi:10.1038/s41598-018-30053-0)
Supplement: Supplementary file 1 — Tables S1–5 and Figure S1 [file 41598_2018_30053_MOESM1_ESM.docx]

**Synthetic colloid resuscitation in severely injured patients: analysis of a nationwide trauma registry (TraumaRegister DGU)**

Peter Hilbert-Carius, M.D., Ph.D., DEAA^1^, Daniel Schwarzkopf, M.Sc.^2^, Konrad Reinhart, M.D., Ph.D.^2,3^, Christiane S. Hartog, M.D., Ph.D.^3,4^, Rolf Lefering, Ph.D.^5^, Michael Bernhard, M.D., Ph.D., MHBA^6^, and Manuel F. Struck, M.D.^7^

^1^Department of Anesthesiology, Intensive Care and Emergency Medicine, Pain Therapy; Bergmannstrost Hospital Halle, Germany

^2^Center for Sepsis Control and Care, Jena University Hospital, Jena, Germany

^3^Department of Anesthesiology and Intensive Care Medicine, Jena University Hospital, Jena, Germany

^4^Klinik Bavaria Kreischa, Kreischa, Germany

^5^Institute of Research in Operative Medicine (IFOM); University Witten-Herdecke, Campus Cologne Merheim, Cologne, Germany

^6^Emergency Department; University Hospital Düsseldorf, Germany

^7^Department of Anesthesiology and Intensive Care Medicine; University Hospital Leipzig, Germany

P.H-C.: peter.hilbert@bergmannstrost.de, D.S.: [daniel.schwarzkopf@med.uni-jena.de](mailto:daniel.schwarzkopf@med.uni-jena.de), K.R.: [konrad.reinhart@med.uni-jena.de](mailto:konrad.reinhart@med.uni-jena.de), C.S.H.: [christiane.hartog@med.uni-jena.de](mailto:christiane.hartog@med.uni-jena.de), R.L.: [rolf.lefering@uni-wh.de](mailto:rolf.lefering@uni-wh.de), M.B.: [michelbernhard@gmx.de](mailto:michelbernhard@gmx.de), M.S.: [manuelstruck@web.de](mailto:manuelstruck@web.de);

Corresponding author:

Manuel F. Struck, M.D.

Department of Anesthesiology and Intensive Care Medicine

University Hospital Leipzig

Liebigstr. 20, 04103 Leipzig, Germany

Phone: +49 341 9717700, Fax: +49 341 9717709

E-Mail: manuelstruck@web.de

Suppl. Table 1: Multiple logistic regression model using generalized estimating equations with an exchangeable covariance matrix for outcome renal replacement therapy

| Predictors | OR | 95% CI | | P-value |
| --- | --- | --- | --- | --- |
|  |  |  |  |  |
| Colloid dosage groups: (reference: Crystalloids only) |  |  |  | 0.033 |
| ≤ 500 ml colloids | 1.17 | 0.97 | 1.42 | 0.105 |
| 500-1,000 ml | 1.1 | 0.9 | 1.34 | 0.365 |
| >1,000 ml | 1.42 | 1.11 | 1.82 | 0.006 |
| RISC II: Abbreviated Injury Scale worst injury (reference: 2) |  |  |  | <0.001 |
| 3 | 1.11 | 0.71 | 1.75 | 0.648 |
| 4 | 1.69 | 1.06 | 2.70 | 0.027 |
| 5 | 2.61 | 1.61 | 4.24 | <0.001 |
| 6 | 1.41 | 0.54 | 3.70 | 0.484 |
| RISC II: Abbreviated Injury Scale second-worst injury (reference: 0-2) |  |  |  | <0.001 |
| 3 | 1.65 | 1.42 | 1.93 | <0.001 |
| 4 | 2.43 | 1.94 | 3.05 | <0.001 |
| 5-6 | 2.05 | 1.55 | 2.7 | <0.001 |
| RISC II: Abbreviated Injury Scale head injury (reference: 0-2) |  |  |  | <0.001 |
| 3-4 | 0.75 | 0.64 | 0.88 | <0.001 |
| 5-6 | 0.41 | 0.31 | 0.52 | <0.001 |
| RISC II: Age (reference: <55) |  |  |  | <0.001 |
| 55-59 | 1.97 | 1.61 | 2.42 | <0.001 |
| 60-64 | 2.52 | 2.04 | 3.12 | <0.001 |
| 65-69 | 2.58 | 1.97 | 3.36 | <0.001 |
| 70-74 | 2.73 | 2.15 | 3.46 | <0.001 |
| 75-79 | 3.44 | 2.70 | 4.38 | <0.001 |
| 80-84 | 3.11 | 2.41 | 4.01 | <0.001 |
| >84 | 2.20 | 1.64 | 2.94 | <0.001 |
| RISC II: Gender (male/missing values vs. female) | 1.76 | 1.56 | 1.99 | <0.001 |
| RISC II: Pre-trauma American Society of Anesthesiologists Score (reference: 1-2) |  |  |  | 0.016 |
| 3 or missing values | 1.13 | 0.97 | 1.32 | 0.129 |
| 4 | 2.15 | 1.23 | 3.78 | 0.007 |
| RISC II: Mechanism (Penetrating vs. blunt/missing values) | 0.65 | 0.48 | 0.87 | 0.004 |
| RISC II: Motor function (reference: normal) |  |  |  | 0.07 |
| Directed or missing values | 0.90 | 0.77 | 1.05 | 0.191 |
| Non-directed | 0.91 | 0.67 | 1.23 | 0.535 |
| None | 1.09 | 0.91 | 1.32 | 0.355 |
| RISC II: Pupil reactivity (reference: brisk) |  |  |  | 0.714 |
| Sluggish or missing values | 1.05 | 0.88 | 1.26 | 0.565 |
| Fixed | 0.98 | 0.76 | 1.26 | 0.856 |
| RISC II: Pupil size (reference: normal) |  |  |  | 0.767 |
| Anisocoria or missing values | 1.02 | 0.85 | 1.23 | 0.816 |
| Both dilated | 1.1 | 0.83 | 1.45 | 0.523 |
| RISC II: Blood pressure (reference: 111-150) |  |  |  | <0.001 |
| 90-110 or missing values | 1.23 | 1.11 | 1.36 | 0.001 |
| <90 | 1.50 | 1.25 | 1.80 | <0.001 |
| RISC II: Cardiopulmonary resuscitation performed | 1.00 | 0.73 | 1.38 | 0.991 |
| RISC II: International normalized ratio (reference: <1.2) |  |  |  | <0.001 |
| 1.2-1.39 | 1.34 | 1.12 | 1.61 | <0.001 |
| 1.4-2.39 or missing values | 1.44 | 1.23 | 1.69 | <0.001 |
| ≥2.4 | 1.83 | 1.46 | 2.3 | <0.001 |
| RISC II: Haemoglobin (reference: ,≥12.0) |  |  |  | <0.001 |
| 7.0-11.9 or missing values | 1.51 | 1.32 | 1.74 | <0.001 |
| <7.0 | 1.69 | 1.35 | 2.11 | <0.001 |
| RISC II: Base deficit (reference: <6.0) |  |  |  | <0.001 |
| 6.0-8.9 or missing values | 1.25 | 1.1 | 1.44 | 0.002 |
| 9.0-14.9 | 2.06 | 1.69 | 2.52 | <0.001 |
| ≥15.0 | 3.08 | 2.19 | 4.32 | <0.001 |
| Transfusion of packed red blood cells | 1.61 | 1.36 | 1.9 | <0.001 |
| Transfusion of more than 10 packs of packed red blood cells | 1.63 | 1.31 | 2.03 | <0.001 |
| Total fluid amount > 4,000 ml | 0.91 | 0.75 | 1.11 | 0.341 |
| Year of treatment > 2011 | 1.43 | 1.21 | 1.69 | <0.001 |

Results of multiple logistic regression model using generalized estimating equations with an exchangeable covariance matrix based on 36,327 cases with ICU stay ≥2 days. Nagelkerke’s R^2^=0.17. OR; odds ratio, CI; confidence interval.

Suppl. Table 2: Multiple logistic regression using generalized estimating equations with an exchangeable covariance matrix for outcome renal failure

| Predictors | OR | 95% CI | | P-value |
| --- | --- | --- | --- | --- |
|  |  |  |  |  |
| Colloid dosage groups: (reference: Crystalloids only) |  |  |  | 0.002 |
| ≤ 500 ml colloids | 1.02 | 0.9 | 1.17 | 0.746 |
| 500-1,000 ml | 1.05 | 0.9 | 1.21 | 0.549 |
| >1,000 ml | 1.32 | 1.12 | 1.57 | 0.001 |
| RISC II: Abbreviated Injury Scale worst injury (reference: 2) |  |  |  | <<0.001 |
| 3 | 1.16 | 0.78 | 1.72 | 0.475 |
| 4 | 1.66 | 1.07 | 2.56 | 0.023 |
| 5 | 2.44 | 1.56 | 3.82 | <<0.001 |
| 6 | 1.86 | 0.9 | 3.87 | 0.095 |
| RISC II: Abbreviated Injury Scale second-worst injury (reference: 0-2) |  |  |  | <<0.001 |
| 3 | 1.6 | 1.4 | 1.83 | <<0.001 |
| 4 | 2.21 | 1.85 | 2.64 | <<0.001 |
| 5-6 | 2.08 | 1.64 | 2.65 | <<0.001 |
| RISC II: Abbreviated Injury Scale head injury (reference: 0-2) |  |  |  | <<0.001 |
| 3-4 | 0.74 | 0.65 | 0.85 | <<0.001 |
| 5-6 | 0.47 | 0.38 | 0.58 | <<0.001 |
| RISC II: Age (reference: <55) |  |  |  | <<0.001 |
| 55-59 | 1.84 | 1.56 | 2.17 | <0.001 |
| 60-64 | 2.56 | 2.16 | 3.02 | <0.001 |
| 65-69 | 2.69 | 2.2 | 3.28 | <0.001 |
| 70-74 | 3.07 | 2.52 | 3.73 | <0.001 |
| 75-79 | 3.91 | 3.29 | 4.64 | <0.001 |
| 80-84 | 4.44 | 3.54 | 5.58 | <0.001 |
| >84 | 4.67 | 3.65 | 5.97 | <<0.001 |
| RISC II: Gender (male/missing values vs. female) | 1.53 | 1.38 | 1.7 | <0.001 |
| RISC II: Pre-trauma American Society of Anesthesiologists Score (reference: 1-2) |  |  |  | <<0.001 |
| 3 or missing values | 1.10 | 0.99 | 1.24 | 0.08 |
| 4 | 2.97 | 1.81 | 4.87 | <0.001 |
| RISC II: Mechanism (Penetrating vs. blunt/missing values) | 0.8 | 0.62 | 1.02 | 0.068 |
| RISC II: Motor function (reference: normal) |  |  |  | 0.064 |
| Directed or missing values | 1 | 0.86 | 1.15 | 0.941 |
| Non-directed | 1.05 | 0.83 | 1.33 | 0.658 |
| None | 1.18 | 1.01 | 1.37 | 0.032 |
| RISC II: Pupil reactivity (reference: brisk) |  |  |  | 0.572 |
| Sluggish or missing values | 1.07 | 0.93 | 1.23 | 0.324 |
| Fixed | 1.08 | 0.88 | 1.31 | 0.442 |
| RISC II: Pupil size (reference: normal) |  |  |  | 0.578 |
| Anisocoria or missing values | 0.98 | 0.85 | 1.14 | 0.791 |
| Both dilated | 1.1 | 0.91 | 1.32 | 0.344 |
| RISC II: Blood pressure (reference: 111-150) |  |  |  | <0.001 |
| 90-110 or missing values | 1.19 | 1.08 | 1.31 | 0.001 |
| <90 | 1.44 | 1.24 | 1.67 | <0.001 |
| RISC II: Cardiopulmonary resuscitation performed | 1.33 | 1.04 | 1.7 | 0.025 |
| RISC II: International normalized ratio (reference: <1.2) |  |  |  | <0.001 |
| 1.2-1.39 | 1.45 | 1.25 | 1.68 | <0.001 |
| 1.4-2.39 or missing values | 1.54 | 1.34 | 1.76 | <0.001 |
| ≥2.4 | 2.08 | 1.71 | 254 | <0.001 |
| RISC II: Haemoglobin (reference: >=12.0) |  |  |  | <0.001 |
| 7.0-11.9 or missing values | 1.43 | 1.28 | 1.59 | <0.001 |
| <7.0 | 1.5 | 1.21 | 1.84 | <0.001 |
| RISC II: Base deficit (reference: <6.0) |  |  |  | <0.001 |
| 6.0-8.9 or missing values | 1.24 | 1.1 | 1.39 | <0.001 |
| 9.0-14.9 | 1.87 | 1.57 | 2.22 | <0.001 |
| ≥15.0 | 2.41 | 1.8 | 3.24 | <0.001 |
| Transfusion of packed red blood cells | 1.49 | 1.28 | 1.74 | <0.001 |
| Transfusion of more than 10 packs of packed red blood cells | 1.6 | 1.33 | 1.92 | <0.001 |
| Total fluid amount > 4,000 ml | 0.91 | 0.77 | 1.06 | 0.252 |
| Year of treatment > 2011 | 1.16 | 0.97 | 1.38 | 0.098 |

Results of multiple logistic regression model using generalized estimating equations with an exchangeable covariance matrix based on 36,327 cases with ICU stay ≥2 days. Nagelkerke’s R^2^=0.17. OR; odds ratio, CI; confidence interval.

Suppl. Table 3: Multiple logistic regression using generalized estimating equations with an exchangeable covariance matrix for outcome multiple-organ-failure

| Predictors | OR | 95% CI | | P-value |
| --- | --- | --- | --- | --- |
|  |  |  |  |  |
| Colloid dosage groups: (reference: Crystalloids only) |  |  |  | <0.001 |
| ≤ 500 ml colloids | 1.36 | 1.24 | 1.5 | <0.001 |
| 500-1,000 ml | 1.39 | 1.27 | 1.53 | <0.001 |
| >1,000 ml | 1.40 | 1.23 | 1.6 | <0.001 |
| RISC II: Abbreviated Injury Scale worst injury (reference: 2) |  |  |  | <0.001 |
| 3 | 1.55 | 1.22 | 1.97 | <0.001 |
| 4 | 2.28 | 1.79 | 2.90 | <0.001 |
| 5 | 3.08 | 2.39 | 3.97 | <0.001 |
| 6 | 3.07 | 2.05 | 4.59 | <0.001 |
| RISC II: Abbreviated Injury Scale second-worst injury (reference: 0-2) |  |  |  | <0.001 |
| 3 | 1.78 | 1.67 | 1.9 | <0.001 |
| 4 | 2.66 | 2.37 | 2.97 | <0.001 |
| 5-6 | 3.11 | 2.58 | 3.74 | <0.001 |
| RISC II: Abbreviated Injury Scale head injury (reference: 0-2) |  |  |  | <0.001 |
| 3-4 | 1.22 | 1.13 | 1.32 | <0.001 |
| 5-6 | 1.31 | 1.16 | 1.47 | <0.001 |
| RISC II: Age (reference: <55) |  |  |  | <0.001 |
| 55-59 | 1.31 | 1.19 | 1.44 | <0.001 |
| 60-64 | 1.6 | 1.45 | 1.76 | <0.001 |
| 65-69 | 1.85 | 1.64 | 2.08 | <0.001 |
| 70-74 | 1.82 | 1.64 | 2.01 | <0.001 |
| 75-79 | 1.97 | 1.74 | 2.23 | <0.001 |
| 80-84 | 2.12 | 1.85 | 2.43 | <0.001 |
| >84 | 2.26 | 1.94 | 2.62 | <0.001 |
| RISC II: Gender (male/missing values vs. female) | 1.35 | 1.27 | 1.43 | <0.001 |
| RISC II: Pre-trauma American Society of Anesthesiologists Score (reference: 1-2) |  |  |  | <0.001 |
| 3 or missing values | 1.01 | 0.91 | 1.23 | 0.864 |
| 4 | 2.48 | 1.66 | 3.7 | <0.001 |
| RISC II: Mechanism (Penetrating vs. blunt/missing values) | 0.72 | 0.64 | 0.82 | <0.001 |
| RISC II: Motor function (reference: normal) |  |  |  | <0.001 |
| Directed or missing values | 1.51 | 1.4 | 1.62 | <0.001 |
| Non-directed | 2.07 | 1.79 | 2.39 | <0.001 |
| None | 2.16 | 1.93 | 2.42 | <0.001 |
| RISC II: Pupil reactivity (reference: brisk) |  |  |  | <0.001 |
| Sluggish or missing values | 1.17 | 1.07 | 1.28 | <0.001 |
| Fixed | 1.41 | 1.24 | 1.6 | <0.001 |
| RISC II: Pupil size (reference: normal) |  |  |  | 0.112 |
| Anisocoria or missing values | 1.04 | 0.96 | 1.14 | 0.332 |
| Both dilated | 1.13 | 1 | 1.27 | 0.055 |
| RISC II: Blood pressure (reference: 111-150) |  |  |  | <0.001 |
| 90-110 or missing values | 1.16 | 1.11 | 1.21 | <0.001 |
| <90 | 1.66 | 1.48 | 1.86 | <0.001 |
| RISC II: Cardiopulmonary resuscitation performed | 1.65 | 1.38 | 1.98 | <0.001 |
| RISC II: International normalized ratio (reference: <1.2) |  |  |  | <0.001 |
| 1.2-1.39 | 1.30 | 1.2 | 1.42 | <0.001 |
| 1.4-2.39 or missing values | 1.39 | 1.26 | 1.52 | <0.001 |
| ≥2.4 | 1.79 | 1.52 | 2.11 | <0.001 |
| RISC II: Haemoglobin (reference: ,>=12.0) |  |  |  | <0.001 |
| 7.0-11.9 or missing values | 1.17 | 1.09 | 1.25 | <0.001 |
| <7.0 | 1.21 | 0.97 | 1.51 | 0.091 |
| RISC II: Base deficit (reference: <6.0) |  |  |  | <0.001 |
| 6.0-8.9 or missing values | 0.94 | 0.87 | 1.02 | 0.141 |
| 9.0-14.9 | 1.61 | 1.41 | 1.85 | <0.001 |
| ≥15.0 | 1.79 | 1.36 | 2.38 | <0.001 |
| Transfusion of packed red blood cells | 1.59 | 1.46 | 1.73 | <0.001 |
| Transfusion of more than 10 packs of packed red blood cells | 1.46 | 1.25 | 1.7 | <0.001 |
| Total fluid amount > 4,000 ml | 1.08 | 0.97 | 1.19 | 0.062 |
| Year of treatment > 2011 | 1.25 | 1.04 | 1.49 | 0.018 |

Results of multiple logistic regression model using generalized estimating equations with an exchangeable covariance matrix based on 36,327 cases with ICU stay ≥2 days. Nagelkerke’s R^2^=0.32. OR; odds ratio, CI; confidence interval.

Suppl. Table 4: Multiple logistic regression using generalized estimating equations with an exchangeable covariance matrix for outcome hospital mortality

| Predictor | OR | 95% CI | | P-value |
| --- | --- | --- | --- | --- |
|  |  |  |  |  |
| Colloid dosage groups: (reference: Crystalloids only) |  |  |  | 0.594 |
| ≤ 500 ml colloids | 0.96 | 0.86 | 1.07 | 0.473 |
| 500-1,000 ml | 0.99 | 0.87 | 1.12 | 0.829 |
| >1,000 ml | 1.06 | 0.91 | 1.24 | 0.485 |
| RISC II: Abbreviated Injury Scale worst injury (reference: 2) |  |  |  | <0.001 |
| 3 | 2.23 | 1.60 | 3.11 | <0.001 |
| 4 | 4.03 | 2.88 | 5.63 | <0.001 |
| 5 | 5.31 | 3.69 | 7.65 | <0.001 |
| 6 | 10.31 | 6.28 | 16.93 | <0.001 |
| RISC II: Abbreviated Injury Scale second-worst injury (reference: 0-2) |  |  |  | <0.001 |
| 3 | 1.18 | 1.05 | 1.31 | 0.004 |
| 4 | 1.64 | 1.41 | 1.92 | <0.001 |
| 5-6 | 3.17 | 2.58 | 3.89 | <0.001 |
| RISC II: Abbreviated Injury Scale head injury (reference: 0-2) |  |  |  | <0.001 |
| 3-4 | 1.03 | 0.94 | 1.13 | 0.573 |
| 5-6 | 2.85 | 2.46 | 3.29 | <0.001 |
| RISC II: Age (reference: <55) |  |  |  | <0.001 |
| 55-59 | 1.82 | 1.54 | 2.16 | <0.001 |
| 60-64 | 2.63 | 2.24 | 3.01 | <0.001 |
| 65-69 | 3.29 | 2.82 | 3.83 | <0.001 |
| 70-74 | 4.83 | 4.11 | 5.68 | <0.001 |
| 75-79 | 7.48 | 6.40 | 8.73 | <0.001 |
| 80-84 | 11.66 | 9.92 | 13.70 | <0.001 |
| >84 | 20.51 | 16.86 | 24.94 | <0.001 |
| RISC II: Gender (male/missing values vs. female) | 1.23 | 1.14 | 1.33 | <0.001 |
| RISC II: Pre-trauma American Society of Anesthesiologists Score (reference: 1-2) |  |  |  | <0.001 |
| 3 or missing values | 1.38 | 1.25 | 1.52 | <0.001 |
| 4 | 5.18 | 3.58 | 7.49 | <0.001 |
| RISC II: Mechanism (Penetrating vs. blunt/missing values) | 1.18 | 1.00 | 1.41 | 0.048 |
| RISC II: Motor function (reference: normal) |  |  |  | <0.001 |
| Directed or missing values | 1.43 | 1.30 | 1.58 | <0.001 |
| Non-directed | 2.12 | 1.82 | 2.47 | <0.001 |
| None | 2.63 | 2.32 | 2.98 | <0.001 |
| RISC II: Pupil reactivity (reference: brisk) |  |  |  | <0.001 |
| Sluggish or missing values | 1.38 | 1.26 | 1.50 | <0.001 |
| Fixed | 3.15 | 2.78 | 3.58 | <0.001 |
| RISC II: Pupil size (reference: normal) |  |  |  | <0.001 |
| Anisocoria or missing values | 1.34 | 1.21 | 1.48 | <0.001 |
| Both dilated | 2.49 | 2.20 | 2.82 | <0.001 |
| RISC II: Blood pressure (reference: 111-150) |  |  |  | <0.001 |
| 90-110 or missing values | 1.18 | 1.09 | 1.27 | <0.001 |
| <90 | 1.55 | 1.39 | 1.73 | <0.001 |
| RISC II: Cardiopulmonary resuscitation performed | 2.03 | 1.67 | 2.47 | <0.001 |
| RISC II: International normalized ratio (reference: <1.2) |  |  |  | <0.001 |
| 1.2-1.39 | 1.57 | 1.42 | 1.73 | <0.001 |
| 1.4-2.39 or missing values | 2.04 | 1.83 | 2.28 | <0.001 |
| ≥2.4 | 2.15 | 1.88 | 2.46 | <0.001 |
| RISC II: Haemoglobin (reference: ,>=12.0) |  |  |  | <0.001 |
| 7.0-11.9 or missing values | 1.21 | 1.10 | 1.32 | <0.001 |
| <7.0 | 1.46 | 1.21 | 1.77 | <0.001 |
| RISC II: Base deficit (reference: <6.0) |  |  |  | <0.001 |
| 6.0-8.9 or missing values | 1.24 | 1.14 | 1.35 | <0.001 |
| 9.0-14.9 | 1.75 | 1.50 | 2.04 | <0.001 |
| ≥15.0 | 3.01 | 2.33 | 3.87 | <0.001 |
| Transfusion of packed red blood cells | 1.35 | 1.19 | 1.54 | <0.001 |
| Transfusion of more than 10 packs of packed red blood cells | 1.74 | 1.50 | 2.02 | <0.001 |
| Total fluid amount > 4,000 ml | 0.89 | 0.79 | 1.00 | 0.058 |
| Year of treatment > 2011 | 1.11 | 1.00 | 1.24 | 0.059 |

Results of multiple logistic regression model using generalized estimating equations with an exchangeable covariance matrix based on 48,443 cases. Nagelkerke’s R^2^=0.56.

OR; odds ratio, CI; confidence interval.

Suppl. Table 5: Organ failure in synthetic colloid dosage subgroups

|  | Rates: n (%) | Adjusted OR^a^ | 95% CI | | P-value |
| --- | --- | --- | --- | --- | --- |
| Respiratory |  |  |  |  | <0.001 |
| Crystalloids only (reference) | 3,609 (18.0%) | 1 |  |  |  |
| Synthetic colloids ≤ 500 ml | 1,537 (25.7%) | 1.33 | 1.22 | 1.45 | <0.001 |
| Synthetic colloids 500-1,000 ml | 1,277 (26.6%) | 1.26 | 1.15 | 1.39 | <0.001 |
| Synthetic colloids >1,000 ml | 1,750 (32.1%) | 1.31 | 1.17 | 1.48 | <0.001 |
| Coagulation |  |  |  |  | <0.001 |
| Crystalloids only (reference) | 1,605 (8.0%) | 1 |  |  |  |
| Synthetic colloids ≤ 500 ml | 730 (12.2%) | 1.29 | 1.16 | 1.44 | <0.001 |
| Synthetic colloids 500-1,000 ml | 662 (13.8%) | 1.28 | 1.13 | 1.45 | <0.001 |
| Synthetic colloids >1,000 ml | 1,069 (19.6%) | 1.36 | 1.17 | 1.57 | <0.001 |
| Liver |  |  |  |  | 0.349 |
| Crystalloids only (reference) | 278 (1.4%) | 1 |  |  |  |
| Synthetic colloids ≤ 500 ml | 141 (2.4%) | 1.12 | 0.88 | 1.41 | 0.356 |
| Synthetic colloids 500-1,000 ml | 153 (3.2%) | 1.15 | 0.91 | 1.46 | 0.24 |
| Synthetic colloids >1,000 ml | 302 (5.5%) | 1.26 | 0.98 | 1.62 | 0.07 |
| Cardiovascular |  |  |  |  |  |
| Crystalloids only (reference) | 4,279 (21.3%) | 1 |  |  |  |
| Synthetic colloids ≤ 500 ml | 1,854 (30.9%) | 1.35 | 1.24 | 1.47 | <0.001 |
| Synthetic colloids 500-1,000 ml | 1,624 (33.8%) | 1.38 | 1.26 | 1.51 | <0.001 |
| Synthetic colloids >1,000 ml | 2,287 (42.0%) | 1.42 | 1.28 | 1.58 | <0.001 |
| Central nervous system |  |  |  |  | 0.004 |
| Crystalloids only (reference) | 4,296 (21.4%) | 1 |  |  |  |
| Synthetic colloids ≤ 500 ml | 1,529 (25.5%) | 1.16 | 1.04 | 1.31 | 0.01 |
| Synthetic colloids 500-1,000 ml | 1,269 (26.4%) | 1.21 | 1.08 | 1.36 | 0.002 |
| Synthetic colloids >1,000 ml | 1,498 (27.5%) | 1.1 | 0.91 | 1.33 | 0.331 |

Results based on 36,320 cases with ICU stay ≥2 days.

a: Adjusted odds ratios result from multiple logistic regression analyses using generalized estimating equations with an exchangeable covariance matrix controlling for the following covariates: Revised Injury Severity Classification score, version 2, variables (Abbreviated Injury Scale worst injury, second-worst injury, head injury, age, gender, mechanism (penetrating vs. blunt), motor function, pupil reactivity, blood pressure, cardiopulmonary resuscitation, International Normalized Ratio, haemoglobin, base deficit; all variables categorized), red blood cell transfusion, transfusion of more than 10 packs of red blood cells, infusion of more than 4,000 ml fluids, treatment until/after 2011.

**Figure S1**


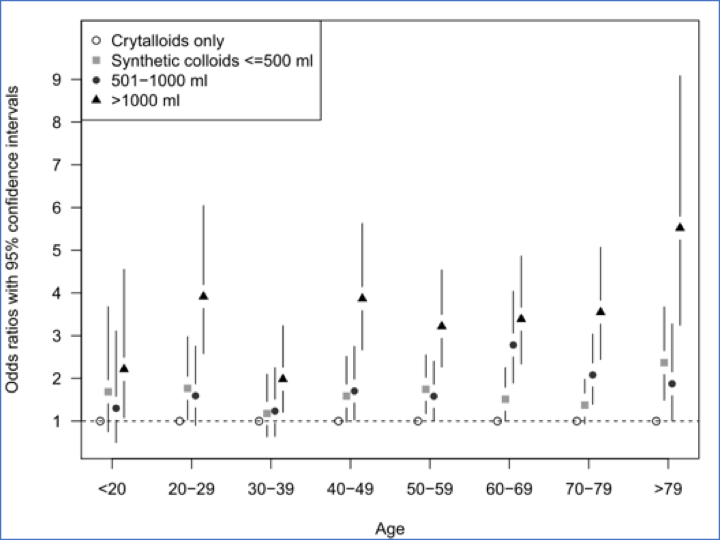


**Figure S1**: Effect of synthetic colloid dosage stratified by age groups. There is no significant difference in the effect of syntethic colloid dosage between age groups (test of interaction between colloid dosage and age group in logistic regression: p=0.383)
